# Diagnostic challenge of malignant rhabdoid tumor presenting as a forehead mass in an infant: A case report

**DOI:** 10.1016/j.ijscr.2025.111372

**Published:** 2025-04-25

**Authors:** Haya El Merkabaoui, Tarek El Hachem, George Greige, Charlie Joe Layoun, Kareem Makkawi, Amir Ibrahim

**Affiliations:** a Department of Plastic and Reconstructive Surgery, American University of Beirut Medical Center, Beirut, Lebanon; b Department of Medicine, American University of Beirut, Beirut, Lebanon

**Keywords:** Malignant extrarenal Rhabdoid tumor, Congenital, Neonate, Pediatric, Head and neck tumors, Case report

## Abstract

**Introduction:**

Rhabdoid tumors (RT) are rare, aggressive malignancies, primarily affecting infants and typically found in the central nervous system, kidneys, or soft tissues, with rare occurrences in the head and neck. These tumors are associated with poor prognosis due to late-stage diagnosis. This case report presents a rare occurrence of a rhabdoid tumor in the forehead, with disseminated disease, highlighting the importance of considering RT in the differential diagnosis for forehead masses to improve outcomes.

**Case presentation:**

We report the case of an infant initially misdiagnosed with a congenital hemangioma, later found to have disseminated malignant rhabdoid tumors (MRTs). Despite the clinical and imaging features suggesting a vascular origin, the persistence of atypical characteristics led to a high suspicion for alternative diagnoses. A collaborative clinical team confirmed MRT after further investigation.

**Clinical discussion:**

Diagnosing MRTs is challenging when they resemble common conditions, such as vascular tumors. In this case, the misdiagnosis as a congenital hemangioma was corrected due to the clinical team's persistence and awareness of atypical features. MRTs are characterized by rapid progression and dissemination, making early detection crucial for effective management.

**Conclusion:**

This case underscores the need for heightened suspicion of rare malignancies, especially with unusual clinical and imaging presentations. Early diagnosis and a collaborative clinical approach are essential for initiating timely treatment and improving patient outcomes. Maintaining a broad differential diagnosis is vital, particularly in cases of disseminated MRTs, to ensure proper management and better prognosis.

## Highlights

• This is a unique case of aggressive extrarenal rhabdoid tumor in a neonate presenting as a forehead mass with metastases.
• Diagnosis is challenging due to overlap with other pediatric head and neck tumors like hemangiomas and Kaposiform hemangioendotheliomas.
• Multidisciplinary team opted for surgery despite high risk of hemorrhage, followed by palliative chemotherapy.
• Immunohistochemistry (loss of INI1), histopathology, and molecular testing confirmed malignant extrarenal rhabdoid tumor.
• Despite treatment, the patient died of organ failure 15 days post-surgery, underscoring poor prognosis of metastatic rhabdoid tumors.

## 1 Introduction

Rhabdoid tumor [RT] is a rare and highly aggressive tumor, most often seen in children between 1 and 4 years of age. It primarily arises in the CNS, as atypical teratoid/RT [AT/RT], kidney [RTK], but can also develop, rarely, extra-renally, such as in the skin, lung, liver, bladder or uterus [1].

Rhabdoid tumor is an uncommon malignancy with a US incidence rate of 0.29 per million in children for renal RTs, 0.89 per million for CNS RTs and 0.32 per million for RTs in other sites [2]. They have a similar genetic mutation in the *SMARCB1*/*hSNF5/INI-1 tumor suppressor* gene, found on chromosome 22q11, either somatic or inherited [2]. This gene has a role in transcriptional regulation [3,4].

This tumor is usually diagnosed at late stages, often stage 3 or higher, and thus has a poor prognosis. In a systematic review of the literature, Metastatic RT was present at diagnosis in more than half the patients (57 %) who had a survival of 2.3 % [5]. Treatment of RTs involve combination therapy of surgery, chemotherapy and radiation, depending on the stage and grade of the tumor [1]. However, reported survival rates for children with metastatic MRT range from as little as 5 days to a maximum of 5 months [6,7].

The present study features a special case of Rhabdoid tumor located in the forehead with disseminated features that was managed at our academic institution. We report to the best of our knowledge, on the first few cases of congenital scalp extracranial rhabdoid tumor, with the first one reported in 2012 [8]. The study aims to review the epidemiology, pathophysiology and differential diagnosis of the case. More importantly, this case-report highlights the importance of including rhabdoid tumors in the differential diagnosis of forehead masses, with the need of a multi-disciplinary approach to improve management, enhance patient outcomes and reduce presentation to diagnosis time.

This case-report was written and reviewed in accordance with the 2023 SCARE criteria [9].

## 2 Case presentation

An 11 day-old preterm female, delivered by planned C/section at 33w0d, not requiring any resuscitation, with a birthweight of 2050 g, was transferred to our institution for management of a forehead mass. The mass was associated with other left cheek and right leg masses.

The baby was started on Propranolol with the suspicion of a hemangioma but there was no decrease in the tumor size.

At presentation to our institution, the patient was clinically and hemodynamically stable.

**Fig. 1:**
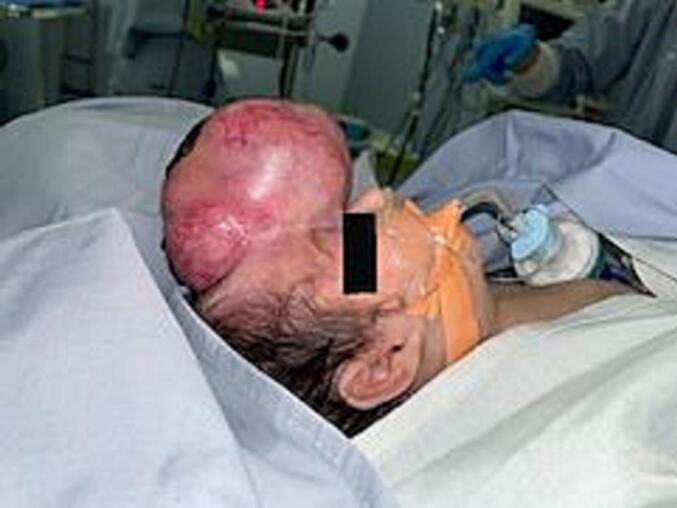
Large frontal pedunculated violaceous mass.

Physical exam showed a large frontal pedunculated violaceous mass (7 × 5 cm) with overlying necrotic area (5 × 3 cm) that minimally bleeds with crying. The mass extended from the left eye, compressing slightly the left nostril (Fig. 1).

The left cheek mass, at the parotid level (7 × 5 cm), was firm, nonmobile with overlying violaceous skin. Another skin color nodular mass was also present over the right lower leg measuring (2 × 3 cm): firm & nonmobile.

Magnetic resonance imaging (MRI) done prior to presentation to our institution showed two large and highly vascular masses, present in the left frontal scalp and left parotid region with central area of necrosis and foci of bleeding, without extension into the brain, osseus structures, cranium, orbits, or airway.

**Fig. 2:**
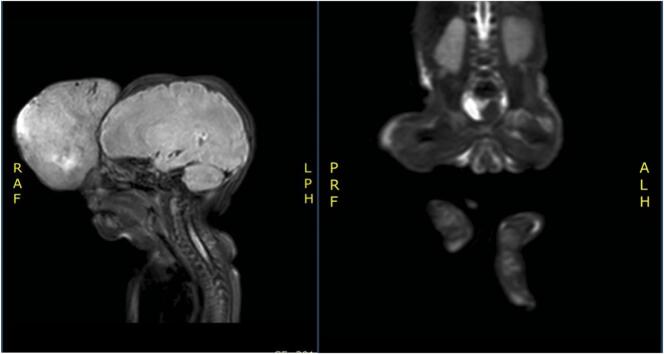
A. MRI of the forehead mass; B. proximal left thigh intramuscular lesion.

During the patient’s stay in our institution, further imaging including MR total body, MRA brain and MRV brain, were performed revealing rapid enlargement of the extracranial tumors. (Fig. 2A) Furthermore, a new intramuscular lesion was noted in the lateral aspect of the left hip/proximal left thigh (Fig. 2B).

Prominent branches of the superficial temporal arteries, supra trochlear and angular arteries wrapping superficially around the tumor were noted, with multiple vessels within this tumor.

Due to the large size, high vascularity and rapid growth of the forehead tumor, a discussion was held with the family addressing both non-operative and operative options, emphasizing the significant risk of bleeding associated with performing a biopsy. The parents elected to proceed with operative management, consisting of a wide surgical excision of the forehead mass followed by application of INTEGRA.

**Fig. 3:**
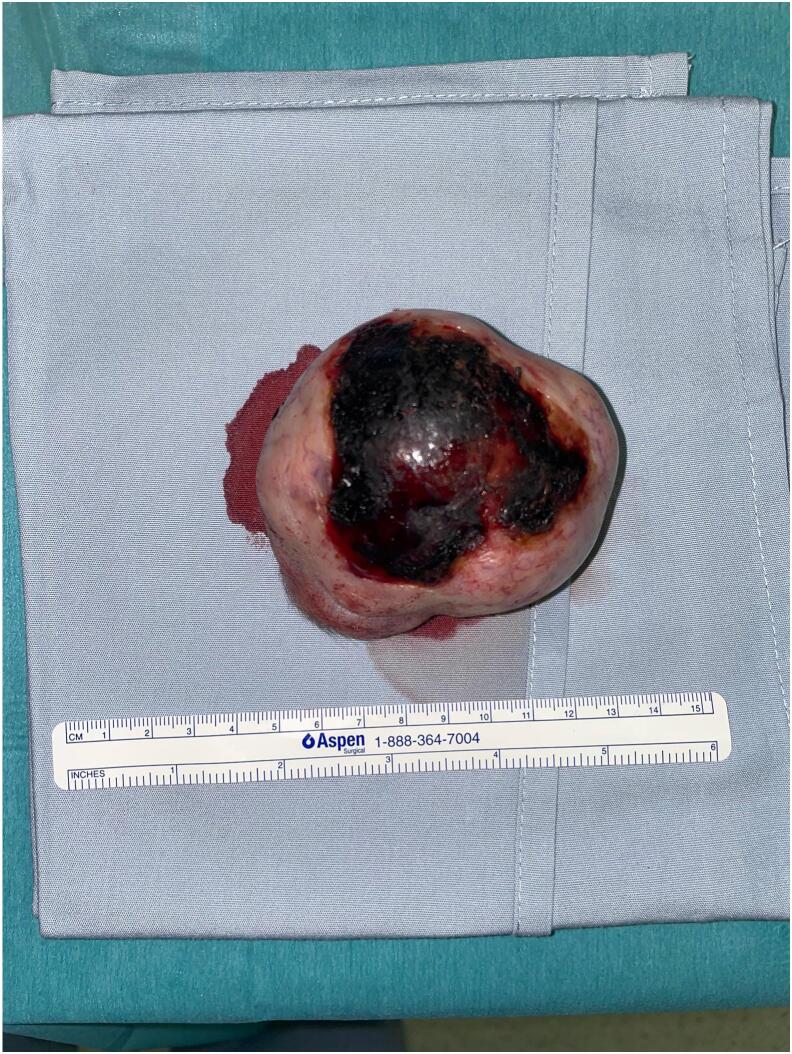
Forehead mass excision intraoperatively showing a highly vascular and necrotic center.

Wide surgical excision of an 8 cm, highly vascular mass over the forehead was performed, ensuring sufficient margin clearance through intraoperative clinical assessment and subsequent pathological analysis (Fig. 3). Pathological evaluation confirmed clear surgical margins (R0 resection). The surgery was complicated by severe bleeding and hemorrhagic shock requiring intraopeartive rescuscitation. Because of high risk of re-bleeding and hemodynamic instability, further surgical intervention was deferred and silver dressing was applied over the defect. The surgical specimen was sent for pathologic and molecular study.

Microscopically, the section showed, subepidermal tumor with dilated vascular channels and mainly round cell tumor infiltrate with prominent nucleoli, scanty cytosplasm, and few avoid. The vascular center was surrounded by prominent necrosis and the nuclei was clear with irregular nuclear membranes.

Immunohistochemical staining on formalin-fixed, paraffin-embedded tissue sections revealed the following: CD99 showed focal and weak positivity, CK AE1/3 was focally positive, WT1 and inhibin were rarely positive, and synaptophysin was retained with some cells exhibiting positive staining. Desmin, CD34, CD45, FLI-1, SOX10, CD1a, SMA, and IN1 showed loss of normal nuclear expression. HMB45 was negative.

Further genetic testing was not conducted due to the family’s financial limitations.

**Fig. 4:**
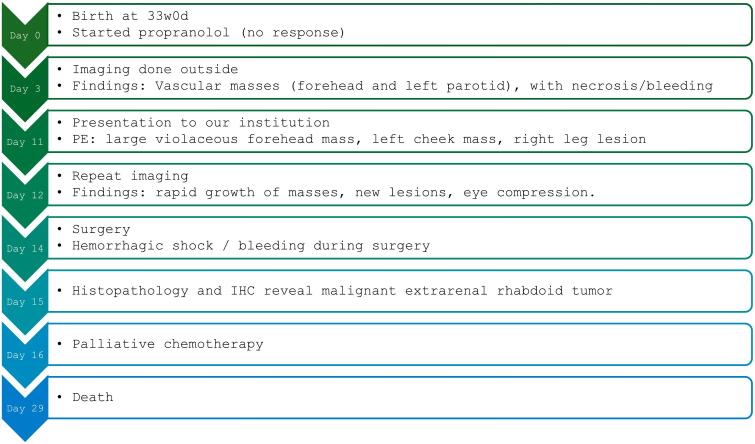
Timeline showing sequence of clinical, diagnostic and clinical intervention.

Tissue examination revealed a final diagnosis of congenital malignant extrarenal rhabdoid tumor. Due to poor prognosis of metastatic rhabdoid tumor and the patient’s critical condition, palliative chemotherapy was decided by the multidisciplinary team and the family. The standard chemotherapy regimen consists of vincristine, dactinomycin, and cyclophosphamide. However, due to concerns about hepatotoxicity, only cyclophosphamide was initiated at a dose of 40 mg/kg, following the rhabdomyosarcoma protocol. Noting no clinical improvement, targeted therapy with larotrectinib was introduced, followed by vinblastine and a reduced dose of methotrexate. Despite these interventions, the tumors progressed rapidly, resulting in the patient’s clinical decline, characterized by worsening respiratory distress, generalized edema, and renal failure. The patient ultimately succumbed to multisystem organ failure 15 days following the surgery (Fig. 4).

## 3 Discussion

The evaluation of pediatric head and neck tumors requires a comprehensive approach to differentiate between various tumor types as the clinical presentation, imaging characteristics, and histopathology of these tumors can often overlap. The initial workup begins with a thorough clinical history and physical examination. Thus, differential diagnosis for this large, neonatal vascular extracranial soft tissue mass includes vascular tumors such as congenital hemangioma and Kaposiform hemangioendothelioma (KHE), neuroblastoma, as well as sarcomas, including angiosarcoma, rhabdomyosarcoma, and fibrosarcoma. Most of these can present similarly, therefore histological and immunohistochemical studies are considered gold standards for the final diagnosis [10]. The infiltrative growth and necrotic feature of the baby’s tumor is usually seen in congenital extrarenal rhabdoid tumors (CERTs), but not with embryonal rhabdomyosarcoma (RMS).

Although congenital hemangioma was initially considered due to the vascular appearance of the tumor, it was later ruled out due to the significant postnatal growth of the tumor and lack of size reduction following beta-blockers administration. Misdiagnosing a rhabdoid tumor as a hemangioma, as seen in our case, is common and has resulted in diagnostic delays of up to eight months [[11], [12], [13]].

Kaposiform hemangioendothelioma (KHE) was ruled out due to the absence of the typical consumptive coagulopathy Kasabach-Merritt phenomenon) and the presence of widespread metastases, which are atypical for KHE [14].

Further imaging studies were essential to narrow the differential diagnosis and assess the extent of the tumor. On MRI, CERTs typically appear as T1 iso-to-hypointense and T2 hyperintense lesions, with central necrosis and heterogeneous enhancement on gadolinium contrast administration. This pattern, while characteristic of CERTs, can also overlap with other tumors like RMS or neuroblastoma.

Histopathologic examination was ultimately needed for diagnosis. CERTs are characterized by undifferentiated round or spindle cells arranged in perivascular patterns with prominent nucleoli and areas of necrosis [15]. The loss of INI1 expression is critical in distinguishing CERTs from other tumors that may exhibit similar histological features, such as RMS or malignant peripheral nerve sheath tumors (MPNSTs), which retain INI1 expression [15]. INI1 loss reflects the inactivation of the SMARCB1 gene, a key component of the SWI/SNF chromatin remodeling complex, leading to uncontrolled tumor growth [16]. Emerging literature suggests that molecular characterization—particularly the identification of SMARCB1 mutations—could allow for targeted therapeutic strategies in the future with ongoing clinical trials exploring targeted agents against pathways associated with SMARCB1 loss [4].

Molecular testing can also be employed to identify SMARCB1 deletions or mutations, which are characteristic of CERTs [16]. Rhabdoid tumors can also be characterized through immunohistochemistry (IHC), with common markers including vimentin and epithelial markers such as cytokeratin. Additional markers like MSA, CEA, SMA, synaptophysin, CD57, and S100 may also be variably expressed, reflecting the tumor’s heterogeneous differentiation [17]. A case series by Fanburg-Smith et al. demonstrated that vimentin and pan-cytokeratin were the most frequently expressed markers in extrarenal rhabdoid tumors of soft tissue [17].

However, in the presented case, further genetic testing was not conducted due to financial constraints.

Arterial embolization was considered to reduce perioperative bleeding but was dismissed due to neonates' low tolerance for radiation and the technical difficulty of catheterizing small pediatric vessels [18]. Surgical resection is the cornerstone of treatment, particularly for tumors like CERTs [1]. This results in a significant forehead defect to cover, based on reconstructive ladder [19]. Skin substitutes like Integra, followed by skin grafting, provide an alternative approach documented in the literature [14,19]. Free flaps, which are on the top of the reconstructive ladder, are considered a last resort for forehead reconstruction due to their prolonged surgical times, which is particularly unsuitable for pediatric patients, and donor site morbidity [20].

While the application of Integra would have been the preferred option, the patient’s critical condition intraoperatively necessitated a time efficient intervention with limited blood loss. As a result, the application of a silver dressing was chosen as the most appropriate immediate intervention.

## 4 Conclusion

This case emphasizes that congenital extrarenal Rhabdoid tumor, although rare, should be included in the differential diagnosis of forehead masses, particularly in neonates presenting with large, rapidly growing, and hypervascular lesions. Careful clinical, radiological, and histopathological assessment is essential for accurate and prompt diagnosis. Due to rhabdoid tumor’s aggressive nature and limited treatment options, early recognition and intervention are critical for improving patient outcomes.

## Author contribution

1. **Haya El Merkabaoui, MD**: Conceptualization, Methodology, Writing – Original Draft, Writing - Review & Editing, Resources, Supervision, Project Administration.
2. **Tarek El Hachem, MD**: Resources, Writing – Original Draft Preparation, Writing - Review & Editing.
3. **George Greige, MD**: Resources, Writing – Original Draft.
4. **Charlie Joe Layoun, BS**: Resources, Writing – Original Draft.
5. **Kareem Makkawi, MD**: Conceptualization, Methodology.
6. **Amir Ibrahim, MD**: Conceptualization, Visualization, Methodology, Supervision.

## Consent

Written informed consent was obtained from the patient’s guardian for the publication of this case report and accompanying images. A copy of the written consent is available for review by the Editor-in-Chief upon request.

## Ethical approval

Our Institutional Review Board (IRB) at the American University of Beirut (AUB) does not require ethical approval for anonymous case reports, as they are not considered human subject research. In this case, we obtained written informed consent from the patient’s legal guardians, both parents.

## Guarantor

Amir Ibrahim, MD.

## Funding

The authors declare no source of funding.

## Registration of research studies

NA.

## Conflict of interest statement

The authors declare no financial and non-financial competing interests.
